# A Scalable, Wide-Angle Metasurface Array for Electromagnetic Energy Harvesting

**DOI:** 10.3390/mi15070904

**Published:** 2024-07-11

**Authors:** Wenping Li, Tao Shen, Binzhen Zhang, Yiqing Wei

**Affiliations:** 1 Faculty of Information Engineering and Automation, Kunming University of Science and Technology, Kunming 650093, China; 2 School of Instrument and Electronics, North University of China, Taiyuan 030051, China; 3 Department of electronics, Xinzhou Normal University, Xinzhou 034000, China

**Keywords:** ambient energy harvesting (AEH), metasurface absorber, rectifier, wide-angle incidence

## Abstract

A metasurface array for electromagnetic (EM) energy harvesting for Wi-Fi bands is presented in this paper; the metasurface array consists of a metasurface unit, a rectifier, and a load resistor. Each row of unit cells in the array is interconnected to form an energy transfer channel, which enables the transfer and concentration of incident power. Furthermore, at the terminal of the channel, a single series diode rectifier circuit and a load resistor are integrated in a coplanar manner. It is used to rectify the energy in Wi-Fi bands and enables DC energy harvesting across the load. Finally, a 5 × 7 prototype of the metasurface array is fabricated and measured for the verification of the rationality of the design. Testing in an anechoic chamber shows that the prototype achieves a 72% RF-DC efficiency at 5.9 GHz when the available incident power is about 7 dBm.

## 1. Introduction

With minimal reflection and transmission coefficients, metamaterial absorbers obtain perfect absorption of incident EM waves at resonant frequencies [1,2,3,4]. However, the energy absorbed by most metamaterial absorbers is not utilized, but is dissipated in the dielectric layer, especially as the number of communication base stations increases [4,5,6,7,8].

In order to keep the large amount of EM energy present in the environment from being wasted, various methods have been proposed. A common approach is to integrate a rectifier into the metasurface absorber, whereby the RF energy is converted to DC. On the basis of this approach, metasurface absorbers can be promising in ambient energy harvesting (AEH). However, it also encounters a practical challenge in that the power density available in the environment is very low, which makes energy harvesting more difficult [9]. As a result, a number of methods have been proposed to improve energy harvesting capacity. One of the simplest methods is to embed a rectifier diode directly inside the metasurface unit cell, thereby achieving the direct conversion of RF energy [10,11,12,13,14,15,16]. This method requires the adjustment of the parameters of the structural unit in such a way that the impedance between the unit and the diode is matched, and is therefore only applicable to some specific resonant structures. In addition, a method has also been proposed to punch holes in the unit cell so as to achieve the transfer and collection of energy at the resonant frequency [17,18,19,20,21,22]. Although effective energy harvesting is achieved, additional layers need to be added to build the impedance matching and rectification network, which greatly increases the difficulty of design and processing. A similar approach has been proposed for energy transfer through EM coupling between multilayer structures, which also requires additional multilayers [23]. To avoid adding multiple layers in terms of transmission and rectification, a method of coplanar integration of rectifier circuits with unit cells has been proposed [24,25,26]. By interconnecting the resonant units and rectifying them at the terminals, the structure of the energy harvester is simplified, the processing difficulty is reduced, and the effective collection of RF energy is achieved. Yet, the metasurface unit size and incident power density need to be larger and higher in order to obtain a high energy harvesting efficiency.

Therefore, a novel scalable, wide-angle incident periodic metasurface energy harvester is proposed in this paper. Unlike the perforation method of transferring energy, we use a metasurface array integrated with rectifier circuits, avoiding the need to add additional layers to the design. Unlike the connection of rectifier diodes integrated in each unit, we constructed an energy transfer channel by interconnecting units in the metasurface array. Thus, the incident power is accumulated, the energy loss caused by the large number of diode rectifiers is reduced, and the energy harvesting efficiency is improved. In summary, the advantages of our design can be summarized as follows: (1) highly efficient energy absorption in the commonly used Wi-Fi frequency band of 5.8 GHz is used for a wider range of applications; (2) wide-angle incidence is used to achieve consistent energy absorption over a wide range of incident angles, further improving its adaptability; (3) interconnected resonant units are used to form energy transfer channels and power accumulation; (4) the single series rectifier circuit at the channel termination is integrated without additional dielectric layers; (5) high energy harvesting efficiency is maintained over a wide range of incident power, being able to achieve a high energy harvesting efficiency of 72% at low input power; (6) a reconfigurable array number is used to accommodate complex environments; and (7) it has a simple structure and easy fabrication.

This paper is organized as follows: In Section 2, we describe the structural design of the metasurface unit and analyze its absorption properties. In Section 3, a single series diode rectifier circuit matching the impedance of the metasurface structure is proposed and its rectification efficiency is simulated and analyzed. In Section 4, a prototype is fabricated and its energy harvesting efficiency is tested and discussed. In Section 5, a conclusion to the article is made.

## 2. Metasurface Array Design and Analysis

**Figure 1:**
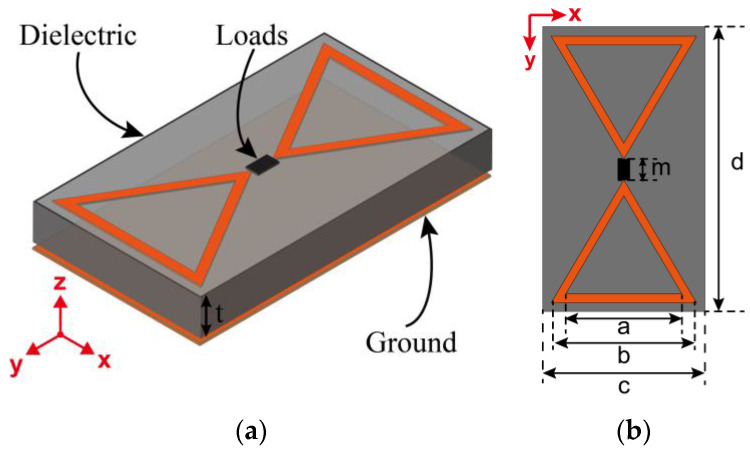
Schematic of the metasurface unit structure. (**a**) Three-dimensional view. (**b**) Top view.

With the energy loss of the dielectric layer, metamaterial absorbers can completely absorb EM waves at the resonant frequency [27,28]. Figure 1 shows the design of a typical sandwich structure of a metasurface array unit. The top layer is structured as two triangular patterns, between which strong EM coupling occurs when the incident EM wave illuminates the surface. The intermediate dielectric is F4B material with a dielectric constant of *ε*_r_ = 2.2, while the loss angle tangent is *tanδ* = 0.0009, which has a thickness of *t* = 3 mm. The idea of choosing such a low loss tangent is that it allows the energy loss on the dielectric layer to be almost negligible. The bottom layer is copper etched on an intermediate dielectric layer, which is 0.035 mm thick—the same as the top layer. As shown in Figure 1b, the inner and outer side lengths of the triangle at the top layer are *a* = 6 mm and *b* = 8 mm, respectively. The metasurface unit is a rectangle with a length of *c* = 27 mm and a width of *d* = 15 mm. The length of the gap between the two triangles is *m* = 2 mm, and such a small spacing induces a strong EM resonance, which, in turn, leads to energy aggregation at the resonance frequency. Next, when a suitable load resistance is attached at the gap, resulting in the unit matching the impedance of the space, the energy of the EM resonance can be fully absorbed via the ohmic losses of the load. Two triangular patterns at the top layer of the metasurface unit are chosen because of the strong EM coupling at the gap, which allows surface currents at the resonant frequency to flow along the two arms of the triangle to the load, and thus, the captured EM energy can be concentrated at the load. In addition, the triangles among units can also induce EM resonance. When the rectifier circuit is terminated at the gap of m, the EM energy captured among units can also flow into the rectifier circuit via the two arms of the triangle, which also improves the ability of capturing EM energy.

**Figure 2:**
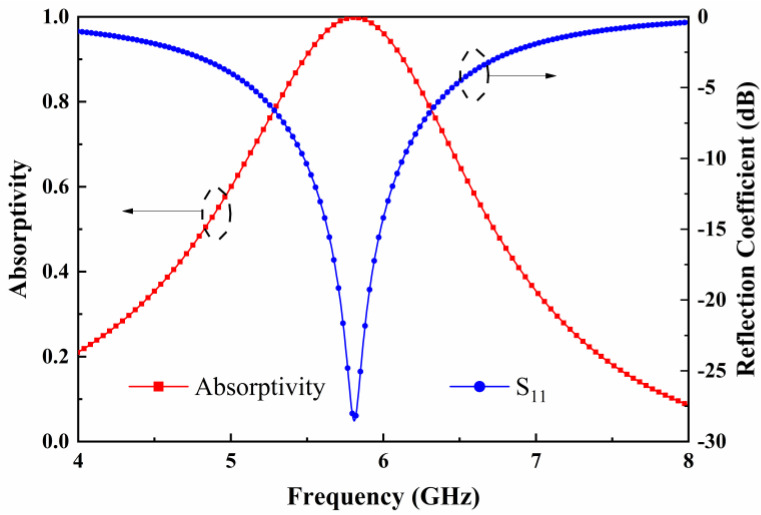
The simulated absorptivity and reflection coefficient of the metasurface unit as a function of frequency.

Figure 2 shows the absorptivity and reflection coefficient versus frequency curves of the metasurface unit when the direction of the electric field of the incident plane wave is parallel to the *y*-axis. The simulation of the designed metasurface array is performed using the software of CST Microwave Studio 2020. The boundary condition is set as a periodic boundary condition with the Floquet port, and the direction of the incident EM wave is along the −*z* direction. The load resistance terminated at the gap between the two triangular patterns is chosen to be 1000 Ω. It can be seen that at a resonant frequency of 5.8 GHz, the reflectivity of the metasurface unit to the incident EM wave is close to 0, since the reflection coefficient goes below −25 dB. Considering that the bottom layer of the metasurface unit is the metal layer, the transmissivity is 0, which enables the absorptivity of the incident EM wave to be close to 1. In this case, the incident EM energy is completely absorbed by the metasurface unit, while for incident EM waves in other frequencies, the absorptivity of the metasurface decreases sharply. It indicates that at 5.8 GHz, the metasurface unit achieves the perfect absorption of the incident EM wave.

**Figure 3:**
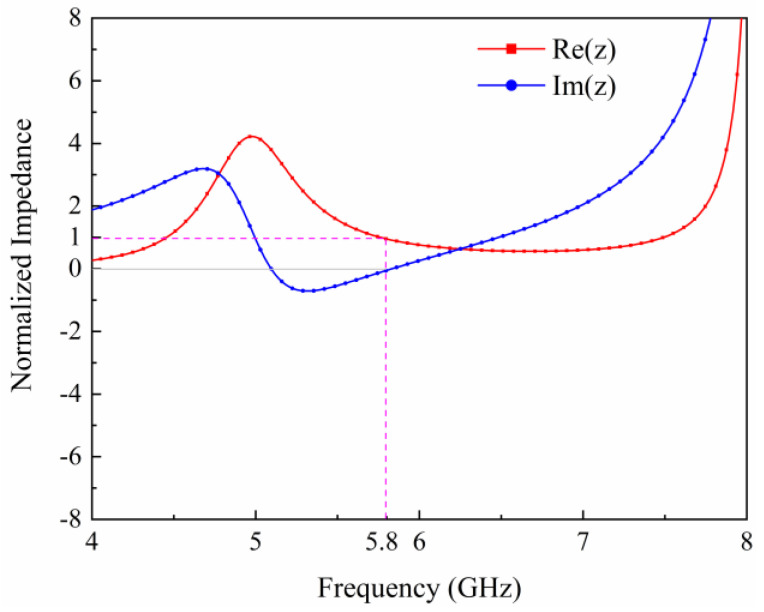
Simulated relative input impedance of the metasurface array unit.

In order to clarify the absorption performance of the metasurface unit, we first analyzed the relative input impedance of the metasurface unit at 5.8 GHz. Figure 3 shows the relative input impedance profile of the metasurface unit at 5.8 GHz. It can be seen that at 5.8 GHz, the real part of the relative input impedance is close to 1, while the imaginary part is close to 0. This means that the metasurface cell has a good impedance match at 5.8 GHz when terminated with a load of 1000 Ω, and therefore has a good absorption performance.

**Figure 4:**
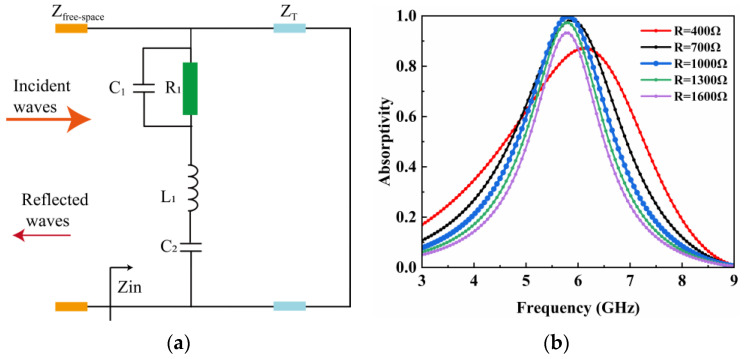
(**a**) Equivalent circuit modeling of the proposed metasurface array. (**b**) Simulated absorptivity of the metasurface unit for different values of the load at the gap.

Second, the absorption performance of the metasurface unit can be elucidated using the transmission line equivalent circuit. Figure 4a depicts the equivalent circuit of the incident EM wave interacting with the metasurface unit. *Z*_free-space_ denotes the free space impedance, which is 377 Ω. The metasurface unit can be modeled using an *RLC* equivalent circuit when irradiated by an incident EM wave. R1 denotes the resistance loaded in the two triangular gap, while *C*_1_ denotes the capacitance at the gap. *L*_1_ and *C*_2_ denote the inductance and capacitance of the top triangle of the unit, respectively. *Z*_T_ denotes the equivalent transmission line of the F4B substrate, which has a characteristic impedance of 377/$\sqrt{\varepsilon_{r}}$ = 254 Ω. Since the bottom layer is entirely covered by copper, it is represented as a short circuit in the transmission line. Figure 4b depicts the variation of the absorptivity of the metasurface resonant unit with frequency for different values of load at the gap. It can be seen that the resonant unit has an absorptivity close to 1 at 5.8 GHz when the value of the load reaches 1000 Ω. This is due to the fact that the equivalent impedance of the metasurface unit matches the free space, and the EM energy is fully incident at the resonant unit, with no reflected EM wave present. In addition, when the load value is changed to 400 Ω, the absorptivity of the resonant unit decreases significantly and the absorption frequency is shifted, which is mainly caused by the impedance mismatch.

**Figure 5:**
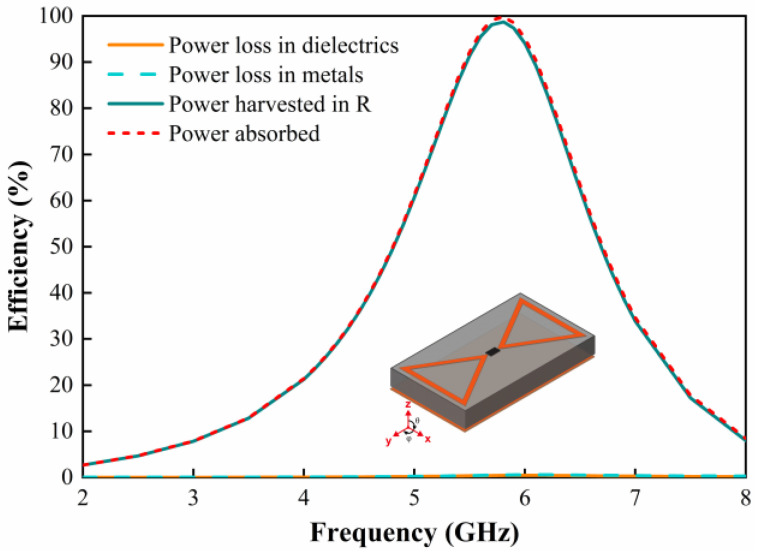
Simulated power absorption, loss, and harvesting efficiencies of the resonant unit when the incident EM wave is illuminated towards the metasurface.

Then, we analyze the absorption performance of the metasurface unit in terms of energy loss in each part of the metasurface unit. When the direction of the E-field of the incident EM wave is parallel to the *y*-axis, the power loss in each part of the metasurface unit is simulated. Figure 5 demonstrates the absorption efficiency of the metasurface unit and the collection efficiency of the dielectric layer, metal, and resistive load. The efficiency of the collection on the metasurface array can be expressed as follows:

(1) $$\eta_{ac}=\frac{P_{ac}}{P_{rad}}$$

where *P*_ac_ denotes the power absorbed by the resistive load, and *P*_rad_ denotes the available input power from the physical area of the metasurface unit. It can be seen that the EM energy absorbed by the metasurface unit is mainly focused on the load, reaching 98.7% at 5.8 GHz. The EM energy concentrated in the dielectric as well as the metal is negligible; therefore, the metasurface array at the resonant frequency can effectively harvest the incident EM energy.

**Figure 6:**
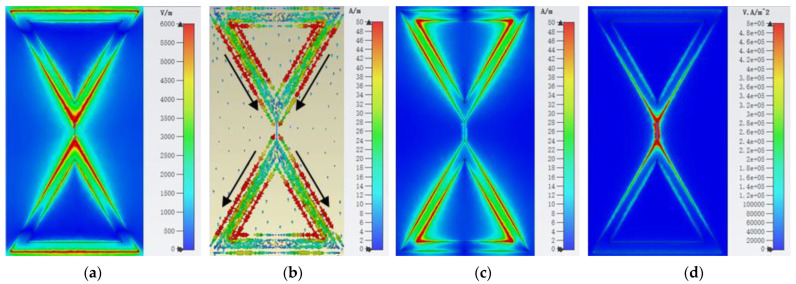
Simulated (**a**) E-field, (**b**) surface current, (**c**) magnetic field, and (**d**) power flow distributions of the resonant unit when the incident EM wave is illuminated to the metasurface at 5.8 GHz.

To further study the resonance and energy absorption mechanisms of the metasurface unit, the E-field, surface current, magnetic field, and power flow distributions of the metasurface unit at 5.8 GHz are analyzed, as shown in Figure 6. From the E-field distribution in Figure 6a, it can be seen that the EM resonance at 5.8 GHz mainly occurs on both sides of the gap between the two triangular patterns. The strong E-field distribution on both sides of the gap indicates a large charge accumulation around the two triangular arms. Influenced by the E-field distribution, the surface currents in the metasurface unit mainly flow along the two triangular arms, as shown in Figure 6b. While the induced current in the bottom layer flows in the opposite direction to the top layer, resulting in a ring current. It then excites the induced magnetic field, which is concentrated around the arms of the triangle, as shown in Figure 6c. Finally, Figure 6d presents the power flow distribution of the metasurface unit at 5.8 GHz. As can be seen, the energy captured by the metasurface unit at the resonant frequency is mainly concentrated on the load at the gap, which is consistent with the simulations in Figure 5.

**Figure 7:**
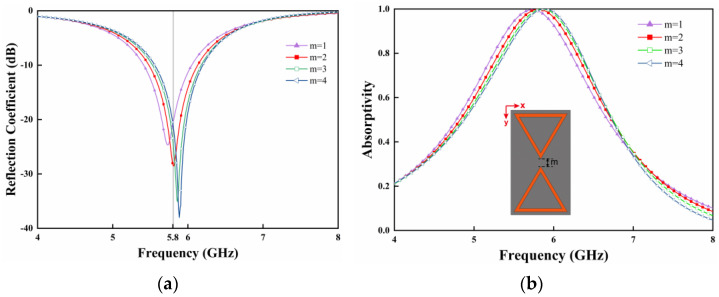
Simulated (**a**) reflection coefficient and (**b**) absorptivity of the metasurface unit for different gap sizes.

Since the resonant strength of the metasurface unit is closely related to the gap m within the structural unit, the absorption performance of the metasurface unit with different gaps is also analyzed. Figure 7a,b show the variation of reflection coefficient, as well as the absorptivity of the metasurface unit with different gaps, respectively. It is observed that the absorptivity of the resonant unit increases with the increase in the gap size. This is because the equivalent capacitance at the gap decreases when the gap in the unit increases, resulting in a higher resonant frequency of the resonant unit. It can be seen from Figure 7a,b that the absorption frequency is shifted with the variation of the gap, while the absorptivity of the resonant unit remains close to 1. Therefore, the desired absorption frequency can be obtained by adjusting the size of the gap.

**Figure 8:**
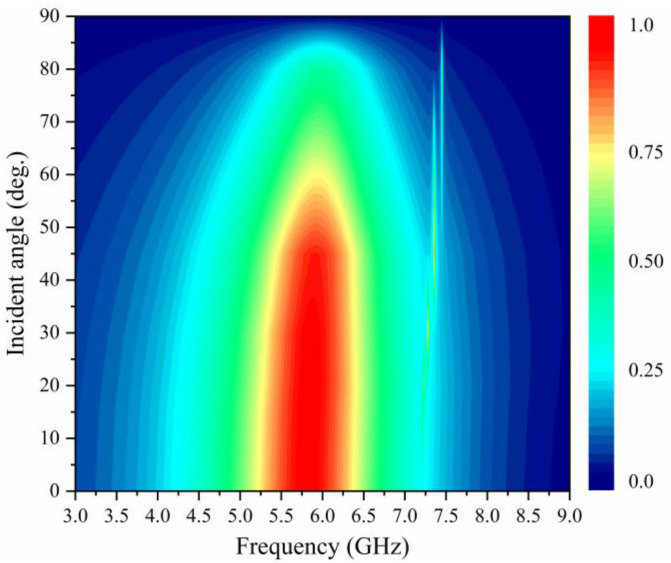
Simulated absorptivity of the metasurface unit at different incidence angles.

For a metasurface energy harvester, the ability to collect energy at different angles is also an important index of its performance. Figure 8 displays the absorptivity of the resonant unit at different angles of incidence, θ, when the incident wave is irradiated onto the surface of the metasurface unit. It can be seen that the absorptivity of the metasurface unit still remains above 0.9 when the incident angle is increased from 0° to 55°. And as the incident angle continues to increase, the absorptivity gradually decreases. This is due to the fact that as the incident angle increases, the intensity of the vertical component of the EM wave decreases, resulting in a weakening of the effective EM resonance on the resonant unit. Overall, the proposed metasurface unit is able to maintain a high absorptivity at wide incidence angles, which greatly improves the environmental adaptability of metasurface energy harvesting.

**Figure 9:**
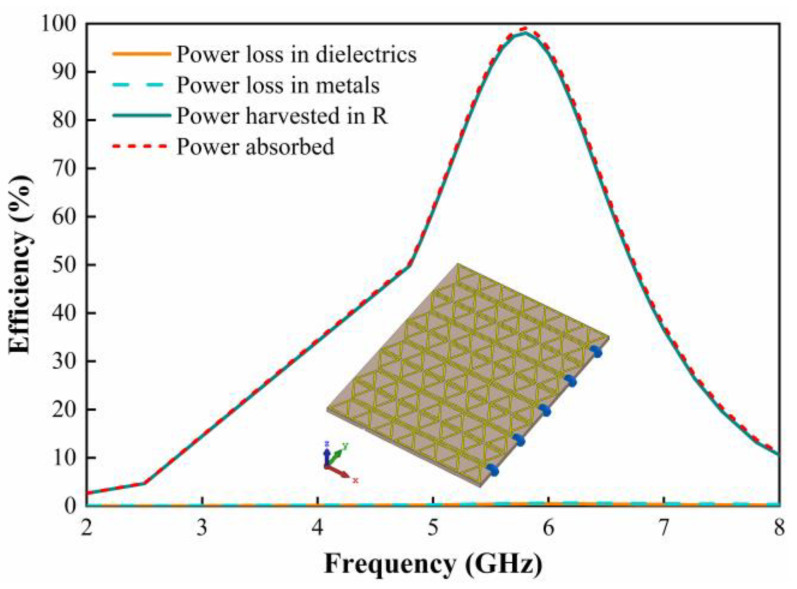
Simulated power absorption, loss, and harvesting efficiencies of the 5 × 7 metasurface array when the incident EM wave is illuminated towards the metasurface.

To achieve DC energy harvesting on the metasurface, it is necessary to replace the load with a rectifier. Considering that the EM energy captured by the metasurface resonant unit is relatively low, the EM energy of multiple units can be aggregated in an array before rectification. Since the metasurface array increases the physical area for EM energy harvesting, it increases the input power of the rectifier, and therefore the DC harvesting of EM energy can be effectively achieved. Figure 9 shows the EM energy distribution of the simulated 5 × 7 metasurface array. The seven resonant units in each column are interconnected to form a unity, and the physical area for EM energy collection is effectively enlarged. The load of 1000 Ω is terminated at the end of each column to harvest the EM energy captured from the seven units. As can be seen in Figure 9, the EM energy on the metasurface array is almost completely absorbed at 5.8 GHz. The efficiency of the EM energy collected on the load reaches 98%, while the energy loss on other dielectrics and metal layers is close to 0. It demonstrates the feasibility of EM energy harvesting by means of the metasurface array.

## 3. Rectifier Circuit Design

**Figure 10:**
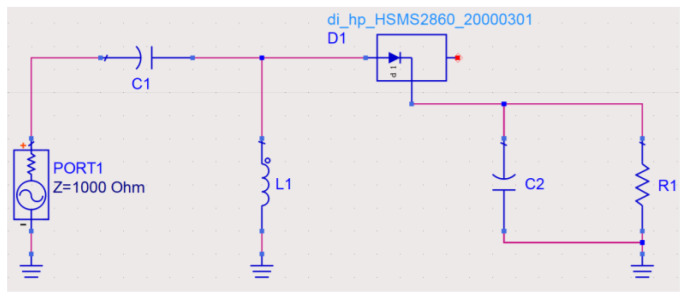
Schematic diagram of the single series diode rectifier circuit proposed in ADS. Here, *C*_1_ = 0.2 pF, *C*_2_ = 1 pF, *L*_1_ = 3.9 nH, *R*_1_ = 2000 Ω.

The rectifier is a crucial part of the AC-DC energy conversion of the metasurface energy harvester and can be constructed with a single series diode, a single shunt diode, or a diode bridge [29,30,31,32,33]. Although the single shunt diode rectifier, the voltage doubler rectifier, and the Greinacher rectifier all have high rectification efficiencies, simulations in the Advanced Design System (ADS) with a harmonic balance solver show that the single series diode rectifier topology has the highest RF-DC efficiency at low input RF power. Therefore, the single series diode rectifier topology is chosen, as shown in Figure 10. It consists of an impedance matching network, a rectifier diode, a capacitor of 1 pf, and a load resistor. A Schottky diode Avago HSMS-2860 with a low turn-on voltage is selected for the rectifier diode to improve RF-DC efficiency at low input power. The impedance matching network consists of a capacitor *C*_1_ and an inductor *L*_1_, which are used to efficiently transfer the EM energy at the gap of the unit to the input of the rectifier diode. *R*_1_ is a load resistor and *C*_2_ is a capacitor for smoothing the waveform and storing the DC supply.

**Figure 11:**
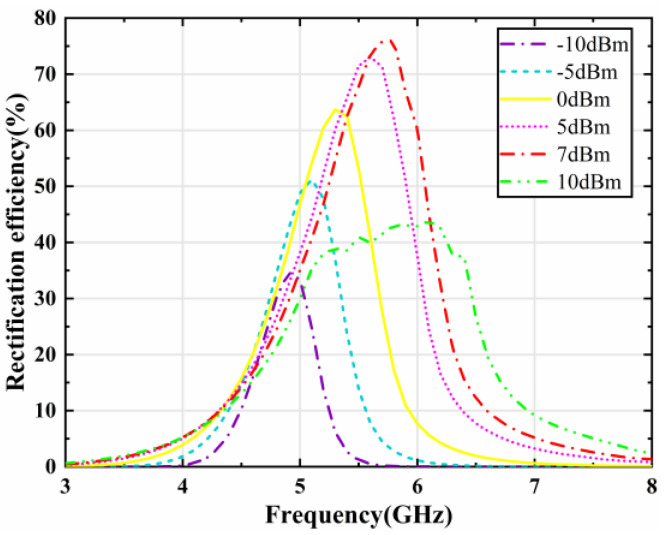
Simulated rectifying efficiency of the rectifier circuit as a function of frequency for different input powers.

Then, the designed rectifier circuit is simulated and optimized via a harmonic balance (HB) solver of the ADS. Figure 11 presents the rectification efficiency versus frequency curves for the rectifier circuit at different input power levels. The rectification efficiency can be expressed as follows:

(2) $$\eta_{dc}=\frac{P_{dc}}{P_{ac}}$$

where *P*_dc_ represents the output power over the load of the rectifier circuit. *P*_ac_ represents the incident power of the rectifier circuit. From the graph in Figure 11, the rectification efficiency of the rectifier circuit shows a parabolic variation with increasing frequency, reaching a maximum at the resonance frequency of 5.8 GHz. Moreover, the rectifying efficiency of the single series diode rectifier reaches a maximum of 76% at 5.8 GHz when the incident power comes to 7 dBm.

**Figure 12:**
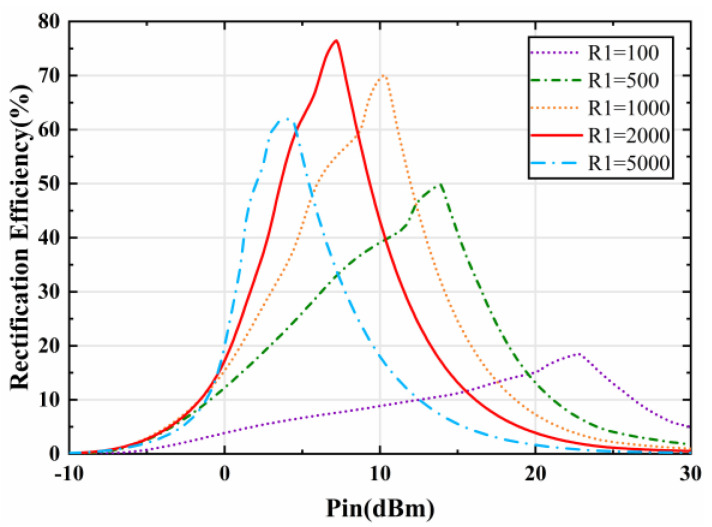
Simulated rectifying efficiency of the rectifier circuit with respect to the variation of input power levels under different loads.

In addition, the rectifying efficiency of the rectifier circuit is analyzed with respect to the input power and load resistance, as shown in Figure 12. It can be seen that when the resonant frequency reaches 5.8 GHz, the rectification efficiency of the rectifier circuit increases with the growth of the input power and then decreases rapidly after reaching the maximum value. Due to the nonlinear effect of the rectifier diode on the input power and load resistance, the input power corresponding to the maximum efficiency is not the same for different loads. It can also be seen from Figure 12 that the rectifier circuit has a maximum of 76.5% at an input power of 7.2 dBm when the load RL is 2000 Ω.

**Figure 13:**
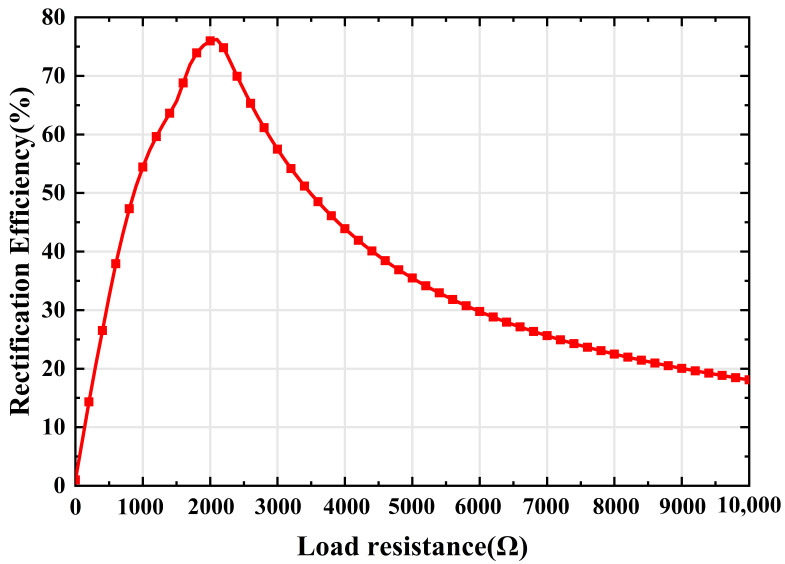
Simulated rectifying efficiency of the rectifier circuit with respect to the variation of load resistance.

Finally, the rectifying efficiency of the rectifier circuit is analyzed with respect to the load impedance at an input power of 7 dBm, as shown in Figure 13. As the load resistance increases, the rectifying efficiency gradually reaches its maximum and then drops continuously. The result is consistent with the maximum shown in Figure 10, and validates the design of the impedance matching network and the rectifier circuit.

## 4. Testing and Discussion

**Figure 14:**
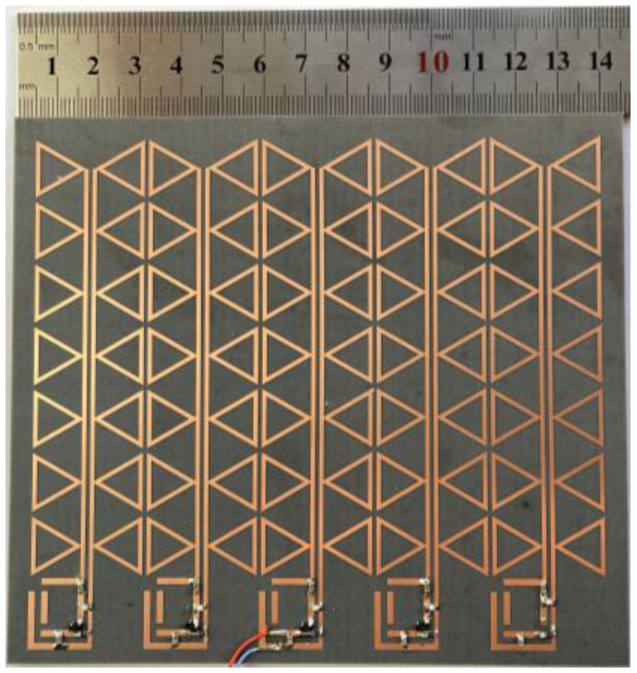
Fabrication of the metasurface array prototype.

Due to the periodic characteristics of the designed metasurface units, the metasurface array exhibits scalable characteristics. The regulation of the size of the metasurface array can be controlled by adjusting the number of units according to different practical needs, such as the limitation of the space of the energy-harvesting environment, which will increase its adaptability in energy harvesting. Figure 14 displays the fabricated structure of a 5 × 7 metasurface array. The structure is plated on the F4B dielectric substrate of 3 mm in thickness with 35 µm-thick copper. The area of the dielectric layer of the prototype is 145 mm × 129 mm, while the area of the underlying copper cladding is 135 mm × 105 mm. The top layer consists of 5 × 7 metasurface units and each row has a single series rectifier circuit integrated into the terminals. Seven metasurface units in each row are connected at the gaps by microstrip lines. This induces the EM energy at the gap of each unit into the terminals and converts them with an integrated rectifier circuit. The seven units connected in the middle row of the metasurface array and a rectifier circuit terminated with it are used as a test, which minimizes the effect of non-uniform coupling and thus maximizes the performance of the periodic metasurface array.

**Figure 15:**
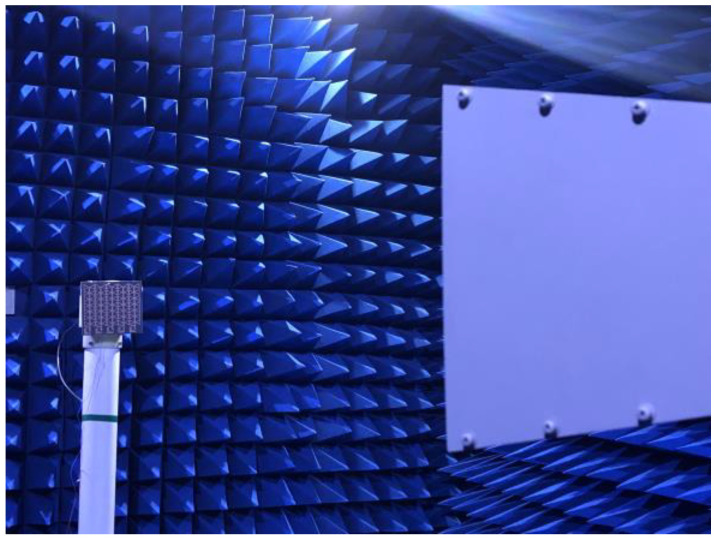
Test photograph of the metasurface array prototype in the anechoic chamber.

The prototype testing of the metasurface array is performed in an anechoic chamber, as shown in Figure 15. At first, the signal generated by the signal generator is transmitted to a standard horn antenna. After that, the horn antenna irradiates the EM waves to the surface of the prototype located at the far-field position. Finally, the DC output voltage across the prototype load is recorded with a digital multimeter. The overall efficiency of the metasurface array energy harvesting is as follows:

(3) $$\eta_{mea.}=\frac{P_{dc}}{P_{in}}$$

(4) $$P_{dc}=\frac{V_{out}^{2}}{R_{load}}$$

(5) $$P_{in}=\frac{G_{t}\cdot P}{{4\pi R}^{2}}\cdot A_{s}$$

where *P*_dc_ represents the output power on the load resistor of the rectifier circuit at the prototype terminals. *P*_in_ represents the overall available incident power absorbed by the surface area of the prototype. *V*_out_ represents the DC output voltage across the rectifier circuit load *R*_load_. *G*_t_ represents the gain of the horn antenna, and *R* represents the distance between the horn antenna and the prototype. *P* represents the power generated by the signal source, and *A_S_* represents the effective receiving aperture of the array.

**Figure 16:**
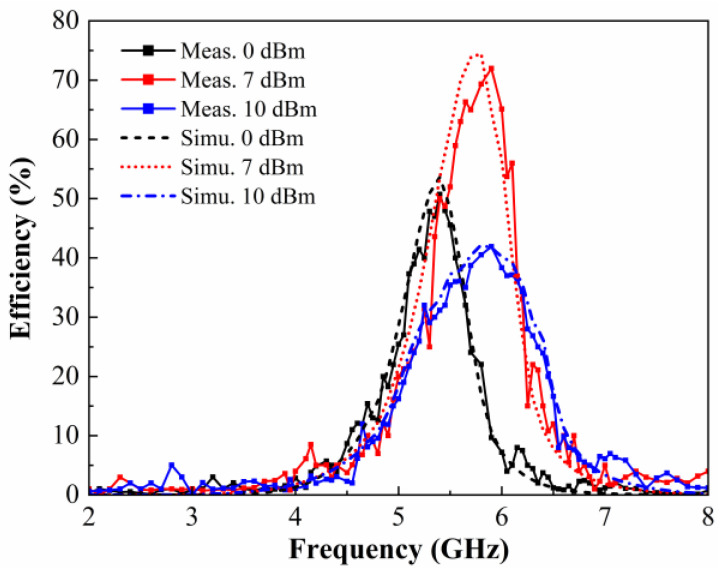
Simulated and measured DC energy harvesting efficiency as a function of frequency for the metasurface array prototype.

Figure 16 shows the simulated and measured RF-DC conversion efficiency versus frequency at different available power levels. For a better comparison between simulation and measurement, the same 5 × 7 array was used for the simulation of the metasurface energy harvesting. It can be seen that the DC conversion efficiency curves of the prototype are characterized by increasing and then decreasing when the incident available power reaches 0 dBm, 7 dBm, and 10 dBm, respectively. Moreover, the energy harvesting efficiency at 7 dBm is significantly higher than the other two curves, reaching 72% at 5.9 GHz. The good agreement between the measurement and simulation results demonstrates the good performance of the fabricated metasurface energy harvester. In addition, the energy harvesting efficiency of the prototypes is lower than the simulation results, and the resonance frequency is also shifted. This is due to the fact that the conversion efficiency of the prototype depends greatly on the rectifying performance of the rectifier circuit. Moreover, non-uniform coupling among metasurface array units with a limited number of units would inevitably cause bias in the test results, as would prototype fabrication.

**Figure 17:**
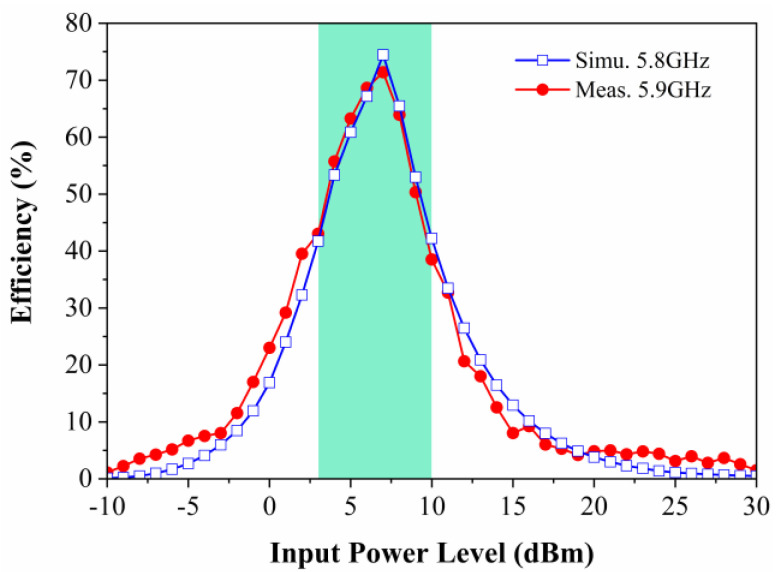
Simulated and measured DC energy harvesting efficiency versus input power when the metasurface array prototype is at 5.9 GHz.

Although the energy harvesting efficiency of the prototype reaches 68% at 5.8 GHz when the incident power is 7 dBm, as shown in Figure 16, the practical resonance frequency of the prototype in the test is 5.9 GHz. In order to accurately measure the energy harvesting efficiency of the prototype under different incident powers, 5.9 GHz is chosen as the test frequency. Figure 17 depicts the simulated and measured conversion efficiency of the metasurface array with respect to the input power available at the surface. The conversion efficiency of the measured metasurface array reaches 72% when the input power is 7 dBm with a load of 2000 Ω. The nonlinear relationship of the rectifier diode with respect to the input power leads to completely different input impedances of the rectifier circuits at different input powers, which results in impedance mismatches, causing a loss of RF energy in the transmission process, as shown in Figure 12. As a result, the DC energy harvesting efficiency of the metasurface energy harvester varies significantly at different incident powers. It can be seen from Figure 17 that the simulated and measured efficiencies remain in good agreement. In addition, the DC energy harvesting efficiency of the metasurface array is higher than 45% when the incident power ranges from 3 to 10dBm, which demonstrates that the designed metasurface energy harvester is able to maintain a high DC energy harvesting efficiency over a wide input power range.

**Figure 18:**
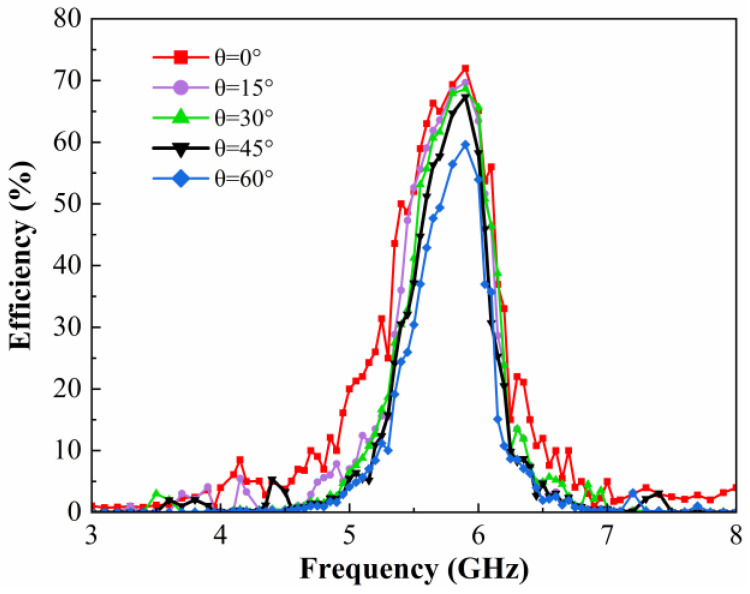
Measured DC energy harvesting efficiencies of the metasurface array prototype at different incidence angles.

To verify the wide-angle absorption capability of the metasurface array, the DC energy harvesting efficiency of the prototype was tested at different incidence angles when the available incident power is 7 dBm, as shown in Figure 18. When the incidence angle is increased from 0° to 45°, the DC collection efficiency at 5.9 GHz stays above 65%, although it decreases slightly. As the incidence angle continues to increase, the DC collection efficiency remains at 60% at 60°. Compared with the absorptivity of AC energy at the metasurface in Figure 8, the measured results coincide with the trend in the simulated results. It illustrates that the designed metasurface energy harvester is able to maintain a high DC energy harvesting efficiency over a wide range of incidence angles.

Finally, the proposed metasurface array is compared with other energy harvesting works reported in the literature. Table 1 lists the performance comparisons in terms of operating frequency, unit size, diode rectifier topology, and DC energy harvesting efficiency, respectively. The proposed metasurface design is capable of achieving a high energy harvesting efficiency in the commonly used 5.8 GHz of the Wi-Fi band. First, the metasurface array adopts the unit’s interconnection method to construct the energy transmission channel, which reduces the loss of energy transmission and improves the collection efficiency of incident energy. Furthermore, the end of each column of transmission channels is terminated using a rectifier topology, so that the energy gathered by each column of units can also be efficiently rectified at low input power. Secondly, the smaller size of the designed unit and only a three-layer structure makes it easier to manufacture, as well as being relatively inexpensive. Finally, it is capable of obtaining high RF-DC collection efficiencies over a wide range of input powers, as shown in Figure 16. Additionally, it features a wide-angle input, which greatly increases its adaptability for energy harvesting. In addition, the scalable characteristic allows it to adjust the number of arrays according to the amount of space in the environment. In summary, the proposed metasurface array has a good energy harvesting performance.

## 5. Conclusions and Future Works

A metasurface array operating in the Wi-Fi band is designed and optimized in this paper. With the units in each row of the metasurface array connected to each other, an EM energy transfer channel is constructed. It enhances the power density and enables the transmission and aggregation of the incident energy, while at the terminal of the transmission channel, a single series diode rectifier circuit is integrated in the coplanar surface to achieve efficient rectification at 5.8 GHz. Then, a 5 × 7 metasurface array prototype is fabricated and tested in an anechoic chamber. The results show that the RF-DC efficiency of the prototype at 5.9 GHz reaches 72% at an incident power of 7 dBm. Furthermore, the metasurface array prototype also maintains a high energy harvesting efficiency over a wide range of input power levels at the resonant frequency. Moreover, the designed metasurface array also has the advantages of simple design, easy processing, and scalable quantity.

Additionally, since the designed metasurface unit is not a centrosymmetric structure, the metasurface array does not maintain a consistent energy harvesting performance for different polarizations, which can lead to application limitations. Therefore, the polarization insensitive property and the wider absorption band are able to enhance the adaptability of energy harvesting, as well as the further research directions in the future.

## Figures and Tables

**Table 1:** Performance comparison of the proposed metasurface array with other works.

Reference	Frequency (GHz)	Diodes to Each Cell	Dimension of Unit Cell (mm)	RF-DC Efficiency (%)
[13]	Single-band 2.25	No	20 × 20 × 6.54 (5 layers)	55%@6 W/m^2^
[15]	Single-band 2.84	Yes	50 × 50 × 3.175 (3 layers)	60%@187.5 μW/cm^2^
[16]	Dual-band 2.4, 5.8	Yes	16 × 15.5 × 1.27 (3 layers)	58%, 51%@0 dBm
[18]	Single-band 2.45	No	24 × 24 × 4.318 (5 layers)	76.8%@0.4 dBm
[20]	Single-band 2.45	Yes	20 × 20 × 4.2 (6 layers)	66.9%@5000 μW/cm^2^
[22]	Single-band 2.45	No	74 × 67 × 4.769 (5 layers)	61%@313 μW/cm^2^
[23]	Single-band 5.8	No	31.7 × 31.7 × 2.07 (5 layers)	55%@35 μW/cm^2^
[24]	Single-band 2.45	Yes	35 × 57.5 × 6.5 (3 layers)	61%@15 dBm
[26]	Dual-band 2.4, 12.6	No	21 × 21 × 3.07 (3 layers)	64%@3 dBm 2.4 GHz, 55%@14 dBm 12.6 GHz
This work	Single-band 5.8	No	27 × 15 × 3.07 (3 layers)	72%@7 dBm

## Data Availability

The original contributions presented in the study are included in the article, further inquiries can be directed to the corresponding author.
